# COVID-19-Associated Pulmonary Aspergillosis in Patients with Acute Leukemia: A Single-Center Study

**DOI:** 10.3390/jof7110890

**Published:** 2021-10-21

**Authors:** Jovan Rajic, Ivana Gmizic, Tara Gunjak, Violeta Milosevic, Nikola Pantic, Nikica Sabljic, Mirjana Mitrovic, Aleksandra Djuric Stefanovic, Ljubica Lazic, Snezana Jovanovic, Ivana Milošević, Aleksandra Barac, Ana Vidovic

**Affiliations:** 1 Clinic for Hematology, University Clinical Center of Serbia, 11000 Belgrade, Serbia; jovadin89@gmail.com (J.R.); tara.gunjak@gmail.com (T.G.); vimar@ptt.rs (V.M.); drnikolapantic@gmail.com (N.P.); nsabljic@gmail.com (N.S.); mirjana.mitrovic777@gmail.com (M.M.); vidana103@gmail.com (A.V.); 2 Clinic for Infectious and Tropical Diseases, University Clinical Center of Serbia, 11000 Belgrade, Serbia; ivana.s.milosevic@mfub.bg.ac.rs (I.M.); aleksandrabarac85@gmail.com (A.B.); 3 Faculty of Medicine, University of Belgrade, 11000 Belgrade, Serbia; aleksandra.djuricstefanovic@gmail.com; 4 Center for Radiology and Magnetic Resonance Imaging, Clinical Center of Serbia, 11000 Belgrade, Serbia; ljbc.lazic@gmail.com; 5 Clinical Center of Serbia, Department of Microbiology, 11000 Belgrade, Serbia; drsnezana.jovanovic@gmail.com

**Keywords:** *Aspergillus*, COVID-19, acute leukemia

## Abstract

Patients with coronavirus disease 19 (COVID-19) have increased susceptibility to secondary respiratory infections including invasive pulmonary aspergillosis (IPA). COVID-19-associated pulmonary aspergillosis (CAPA) is difficult to diagnose and can be associated with increased mortality especially in severe immunodeficiency such as hematological malignancies. Our study evaluates IPA in COVID-19 patients defined as COVID-19-CAPA among patients with acute leukemia (AL). A retrospective single-center study analyzed 46 patients with COVID-19 infection and acute leukemia, admitted to the Clinic for Haematology, Clinical Center of Serbia, Belgrade between the 2 April 2020 and 15 May 2021. During hospitalization, all participants were diagnosed with probable IPA according to the previous consensus definitions. Positive serology and galactomannan (GM) detection values in bronchoalveolar lavage (BAL) and serum were used as microbiological criteria. COVID-19 associated probable IPA was found in 22% (9/41) tested patients, where serum GM and IgM anti-*Aspergillus* antibodies were positive in 12% (5/41) and 10% (4/41) had positive serology for aspergillosis. One patient died while eight recovered during follow-up. Our study showed that COVID-19 might be a risk factor for IPA development in patients with AL. Early diagnosis and prompt treatment are required as reported mortality rates are high.

## 1. Introduction

Patients with primary COVID-19 have increased susceptibility to secondary respiratory infections which may lead to greater severity of illness and may complicate treatments [1]. While most studies have been focusing on COVID-19, fungal co-infections have been neglected. Several studies have been reporting co-infection with *Aspergillus* sp., with an incidence ranking from 19.6% to 33.3% [2]. COVID-19-associated pulmonary aspergillosis (CAPA) is difficult to diagnose and can be associated with increased mortality reaching up to 64.7% [2,3]. Early diagnosis of CAPA is crucial for successful treatment, yet conventional microscopy and culture of respiratory tract sample has only low sensitivity and specificity of around 50% [4]. Among COVID-19 patients, 3.7% were described as immunocompromised [5]. Hematological malignancy was the most frequent cause of immunodeficiency with a 34% mortality risk rate of [6,7,8]. There are insufficient data on the specific types of hematological malignancies in published studies, including the incidence of CAPA in such conditions. In severely immunocompromised patients, CAPA typically involves the lung, and chest computed tomography (CT) may detect lung involvement at an early stage of infection [9]. CT has a significant role in the very early stages of the COVID-19 infection, when the nasopharyngeal swab may still be negative, to eventually place the diagnosis of COVID-19 in patients highly suspicious (i.e., clinical features and exposure history) and set up a prognosis, as well as over the course of the disease for evaluating changes in severity requiring treatment adjustments [10]. Thoracic CT scans may be difficult to assess in patients with ARDS-associated COVID-19 where the diffuse bilateral lung infiltrates may obscure any diagnosis clues for IPA. In this work, we aim to report a series of co-infection of COVID-19 and CAPA in patients with acute leukemia (AL). 

## 2. Materials and Methods 

A prospective single-center study of 46 patients with acute leukemia and COVID-19 co-infection admitted to the University Clinic for Haematology, Clinical Centre of Serbia Belgrade, Serbia, has been performed during the COVID-19 pandemic. Inclusion criteria were: (1) patients with acute leukemia admitted to the Clinic for Heamatologyin Belgrade between 2 April 2020 and 15 May 2021; (2) positive result in the reverse transcription-polymerase chain reaction (RT-PCR) assay for SARS-CoV-2 in respiratory samples (nasopharyngeal swab, tracheal aspirate, bronchial aspirate or bronchoalveolar lavage fluid); (3) and with suspicion of CAPA based on clinical features, thoracic CT scan, serology and galactomannan testing. 

*Aspergillus* infection was classified as possible/probable/proven according to the European Organization for Research and Treatment of Cancer/Invasive Fungal Infections Cooperative Group and the National Institute of Allergy and Infectious Diseases Mycoses Study Group (EORTC/MSG) [11]. For diagnosing probable aspergillosis all four criteria had to be met: (1) *Aspergillus*-positive lower respiratory tract culture; (2) compatible signs and symptoms—one or more of the following: fever refractory to at least three days of appropriate antibiotic therapy, recrudescent fever after a period of defervescence, pleuritic chest pain, pleuritic rub, dyspnea, hemoptysis, worsening respiratory insufficiency despite appropriate antibiotic therapy and ventilatory support; (3) abnormal chest X-ray or computed tomography (CT) scan; (4) either presence of host risk factors—one of the following (neutropenia, underlying hematological or oncological malignancy, glucocorticoid treatment with prednisone equivalent >20 mg/day, congenital or acquired immunodeficiency) or microbiological criterion with *Aspergillus*-positive culture of bronchoalveolar lavage (BAL) fluid without bacterial growth and a positive cytological smear showing branching hyphae. Positive serology and galactomannan (GM) detection values in BAL and serum were added to this algorithm as a microbiological criterion (modified AspICU) [12]. As one of the criteria for probable aspergillosis, we used thoracic CT scan which had to fulfill one of the following four patterns: (1) dense, well-circumscribed lesion(s) with or without a halo sign, (2) air crescent sign, (3) cavity and (4) wedge-shaped and segmental or lobar consolidation. 

Data on patient characteristics and outcomes were extracted from electronic medical records or clinical charts, including age, sex, type and status of hematological malignancy, applied chemotherapy, status of bone marrow aplasia, COVID-19 severity, median time from confirmation of COVID-19 to death outcome, and overall survival form confirmation of COVID-19 to recovery. Diagnosis of leukemia was made on the basis of WHO classification of hemato-poetic tumors [13]. Active chemotherapy was the one applied during the COVID-19 co-infection. COVID-19severity was graded by China Centers for Disease Control and Prevention [14]. Written informed consent was collected from all patients.The study has been approved by institutional reviews board (No 111/9), on 24 March 2021.

Continuous variables were expressed as mean (SD) or median (IQR), while categorical variables are presented as frequencies and percentages. The overall survival (OS) was measured as median length of time from confirmation of COVID-19 to either death or recovery. Simultaneously, we measured the overall survival for AL as a time from the date of diagnosis and date of death by any cause, hospital discharge, or last follow-up, whichever occurred first.

## 3. Results

**Figure 1:**
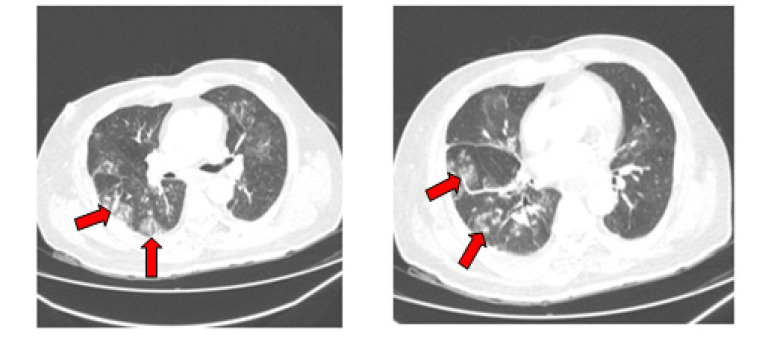
Chest CT scan revealed multiple vessel-related nodular opacities with grand glass halo with central and peripheral distribution, made at the time of COVID-19 diagnosis.

In this study we included 46 patients with diagnosed one of the different types of AL. The mean age was 50 ± 30 years and 54% (*n* = 25) were female. Most frequent acute leukemia was acute myeloid leukemia (*n* = 31, 67%) followed by acute lymphocytic leukemia (*n* = 10, 22%) and acute promyelocytic leukemia (*n* = 5, 11%). Complete remission (CR) of disease was observed in 19 (41%) patients. Eighteen (40%) patients had newly diagnosed leukemia, while the remaining patients had the refractory or relapsed type of AL. Thirty-one (67%) patients were received chemotherapy and 37% were in aplasia at the time of COVID-19 diagnosis. COVID-19 severity was classified as severe/critical in 43% of patients, moderate in 22% and mild in 35%. During follow-up, 14 (30%) died attributable to COVID-19, while 5 patients (11%) died due to hematology malignancy. Median time from confirmation of COVID-19 to death was 13 days (IQR 4–34). Overall survival from confirmation of COVID-19 to recovery was 187 days (QR 62–398). OS due to AL was 402 days. The initial thoracic CT scan was performed in 19 patients (41%) and in 15 cases (32%) it showed signs of bilateral pneumonia, while four cases (8%) showed ground-glass opacities in inferior lung lobes (Figure 1).

Among 32 recovered patients, we performed 18 control thoracic CT scans (56%). CT revealed complete pneumonia regression in 8/32 cases (25%), while in 10/32 cases (31%) regression of pneumonia was incomplete. Serum GM and IgM anti-*Aspergillus* antibodies were performed in 41 patients (89%), where 5 (12%) of them had positive both tests, while4 (10%) had positive serology for aspergillosis. In 9 (22%) patients probable aspergillosis was diagnosed. Among those patients, one died (11%) while eight (89%) are recovered and under follow-up (Table 1).

Overall, 30 patients (65%) received systemic corticosteroids (mainly prednisone and methylprednisolone), 11 patients (24%) received chloroquine, nine patients (20%) received favipiravir, and four (8%) patients received off-labeled tocilizumab. All nine patients diagnosed with probable aspergillosis were treated with voriconazole, loading dose 6 mg/kg twice a day for two doses, followed by 4 mg/kg twice a day for 14 days and continued with maintenance dose of itraconazole 200 mg/day in the next 4 to 10 weeks [15].

## 4. Discussion

Our study showed that patients with AL and COVID-19 have high rates of severe/critical disease and mortality (43% and 30%, respectively). Compared to published studies general population with COVID-19, these rates are much higher (15% and 10%, respectively) [8,16,17]. We found a high incidence of probable aspergillosis (22%) in our cohort of 46 patients which is in agreement with other published data (from 19.6% to 33.3%). These high rates indicate that patients with hematological malignancies and COVID-19 are at a high risk of developing IPA [4,18]. IPA in severely immunocompromised patients typically involves the lung, and CT may detect lung involvement at an early stage of infection [10]. In particular, the halo sign (a micronodule around 1 cm in diameter surrounded by a perimeter of GGO) is regarded to be an early indicator of IPA and the entry criteria for the Global Comparative Aspergillosis Study [11,17]. In our case, 15 of 19 performed CT scans showed bilaterally presence of diffuse patchy GGOs, while in 4/19 cases GGOs were present in inferior lobes. Classic findings of angio-invasive fungal infection (infarct shaped consolidation, cavity, halo signs, mass or nodules) were difficult to assess. Early diagnosis of CAPA is crucial for successful treatment. Detection of GM from bronchoalveolar lavage fluid has been shown as a useful and rapid tool for the identification of IPA in both immunocompromised and immunocompetent patients [18]. Bronchoscopy generates aerosol that causes a substantial risk for staff and patients, and due to thrombocytopenia in patients with AL it was not performed in our cases. Therefore, GM testing is used as a suitable diagnostic assay for IPA. This assay is a sensitive diagnostic marker (70%) in neutropenic patients and it was positive in five our patients with AL [19]. The European Organisation for Research and Treatment of Cancer/Mycoses Study Group (EORTC-MSG) criteria for IPA are appropriate for patients with hematological malignancies including leukemia, and our nine patients (22%) were considered to have probable CAPA based on an abnormal thoracic CT scan and positive results of non-cultures analysis: serum GM or anti-*Aspergillus* IgM Ab, in addition to typical COVID-19 concomitant lesions [13]. All our patients received voriconazole, which is in line with the ECMM (European Organisation for Research and Treatment of Medical Mycology) expert guideline that recommends voriconazole as first-line therapy for suspected and confirmed CAPA in 6–12 weeks duration of treatment and follow-up chest imaging [20]. In our daily practice, administering voriconazole is limited to a hospital setting and the duration of the treatment is 10 to 14 days, which was prolonged with itraconazole for an additional 4 to 10 weeks, respectively. Voriconazole remains the recommended first-line treatment for IPA, but its effectiveness in CAPA remains questionable due to limited number of studies [21]. It has narrow therapeutic window and drug-drug interaction with multiple other drugs including anti-COVID-19 drugs metabolized by CYP3A4 [22]. The optimal duration of treatment remains unknown, but its recommended to include follow-up lung CT imaging to document the resolution of GGOs before the termination of treatment [20]. Eight patients from our study recovered from CAPA and showed improved CT scans of lungs after 12 weeks of treatment. Recommended weekly therapeutic drug monitoring was not available in our routine practice, which is one of the limitations of our study. 

## 5. Conclusions

Our study showed that COVID-19 might be a risk factor for IPA development in patients with AL. Early diagnosis and prompt treatment are required because of the reported high mortality rates. Until then, hematologists, infectious disease specialists, clinical microbiologists and radiologists should be aware of it and perform screening for secondary fungal pulmonary infection especially in immunocompromised patients.

## Figures and Tables

**Table 1:** Main patients’ characteristics, thoracic CT scans comparisons, serology and galactomannan (GM) detection in serum, outcomes.

	Age	Sex	Type of AL	Initial Thoracic CT Scan	Control Thoracic CT Scan	GM Serum	IgM Anti-*Aspergillus* Antibodies	COVID-19 Outcome	Final Outcome
P1 *	55	Female	AML	Widespread GGOs	complete regression	Positive	Positive	Recovered	Alive
P2 *	56	Female	AML	Widespread GGOs	complete regression	Positive	Positive	Recovered	Dead
P3 *	34	Male	APL	Widespread GGOs	complete regression	Negative	Positive	Recovered	Alive
P4 *	58	Male	AML	Widespread GGOs	incomplete regression	Negative	Positive	Recovered	Alive
P5 *	37	Male	AML	N/A^5^	N/A	Negative	Positive	Recovered	Alive
P6 *	58	Male	AML	GGOs in inferior lung lobes	incomplete regression	Negative	Positive	Recovered	Alive
P7 *	27	Female	AML	GGOs in inferior lung lobes	complete regression	Positive	Positive	Recovered	Alive
P8 *	26	Male	ALL	Widespread GGOs	incomplete regression	Positive	Positive	Recovered	Alive
P9 *	53	Male	AML	Widespread GGOs	complete regression	Positive	Positive	Recovered	Alive

Abbreviations: AML, acute myeloid leukemia; APL, acute promyelocytic leukemia; ALL, acute lymphocytic leukemia; GGO, ground-glass opacities; N/A, not available; * P1–9, patients No. 1 to 9.

## Data Availability

The data that support the findings of this study are available on request from the corresponding author I.G. The data are not publicly available since containing information compromise research participant privacy/consent.

## References

[1] Feldman C., Anderson R. (2021). The role of co-infections and secondary infections in patients with COVID-19. Pneumonia.

[2] Alanio A., Dellière S., Fodil S., Bretagne S., Mégarbane B. (2020). Prevalence of putative invasive pulmonary aspergillosis in critically ill patients with COVID-19. Lancet Respir. Med..

[3] Blaize M., Mayaux J., Nabet C., Lampros A., Marcelin A.-G., Thellier M., Piarroux R., Demoule A., Fekkar A. (2020). Fatal invasive aspergillosis and Coronavirus dis-ease in an immunocompetent patient. Emerg. Infect. Dis..

[4] Lai C.-C., Yu W.-L. (2021). COVID-19 associated with pulmonary aspergillosis: A literature review. J. Microbiol. Immunol. Infect..

[5] El-Baba F., Gao Y., Soubani A.O. (2020). Pulmonary Aspergillosis: What the Generalist Needs to Know. Am. J. Med..

[6] CDC COVID-19 Response Team (2020). Preliminary estimates of the prevalence of selected underlying health conditions among patients with Coronavirus Disease 2019-United States, February 12–March 28, 2020. MMWR Morb. Mortal. Wkly. Rep..

[7] Venkatesulu B.P., Chandrasekar V.T., Girdhar P., Advani P., Sharma A., Elumalai T., Hsieh C.E., Elghazawy H.I., Verma V., Krishnan S. (2021). A Systematic Review and Meta-Analysis of Cancer Patients Affected by a Novel Coronavirus. JNCI Cancer Spectr..

[8] Vijenthira A., Gong I.Y., Fox T.A., Booth S., Cook G., Fattizzo B., Martín-Moro F., Razanamahery J., Riches J.C., Zwicker J.I. (2020). Outcomes of patients with hematologic malignancies and COVID-19: A systematic review and meta-analysis of 3377 patients. Blood.

[9] Mitrovic M., Pantic N., Sabljic N., Vucic M., Bukumiric Z., Virijevic M., Pravdic Z., Rajic J., Todorovic-Balint M., Vidovic A. (2021). Acute leukemia and SARS-CoV-2 infection: Clinical characteristics and risk factors for mortality. Leuk Lymphoma..

[10] Hope W., Walsh T., Denning D. (2005). Laboratory diagnosis of invasive aspergillosis. Lancet Infect. Dis..

[11] Hauggaard A., Ellis M., Ekelund L. (2002). Early chest radiography and CT in the diagnosis, management and outcome of invasive pulmonary aspergillosis. Acta Radiol..

[12] Xie X., Zhong Z., Zhao W., Zheng C., Wang F., Liu J. (2020). Chest CT for Typical Coronavirus Disease 2019 (COVID-19) Pneumonia: Relationship to Negative RT-PCR Testing. Radiology.

[13] Donnelly J.P., Chen S.C., Kauffman C.A., Steinbach W.J., Baddley J.W., Verweij P.E., Clancy C.J., Wingard J.R., Lockhart S.R., Groll A.H. (2019). Revision and Update of the Consensus Definitions of Invasive Fungal Disease From the European Organization for Research and Treatment of Cancer and the Mycoses Study Group Education and Research Consortium. Clin. Infect. Dis..

[14] Hamam J., Navellou J.-C., Bellanger A.-P., Bretagne S., Winiszewski H., Scherer E., Piton G., Millon L., Collaborative RESSIF Group (2021). New clinical algorithm including fungal biomarkers to better diagnose probable invasive pulmonary aspergillosis in ICU. Ann. Intensiv. Care.

[15] Arber D.A., Orazi A., Hasserjian R., Thiele J., Borowitz M.J., Le Beau M.M., Bloomfield C.D., Cazzola M., Vardiman J.W. (2016). The 2016 revision to the World Health Organiza-tion classification of myeloid neoplasms and acute leukemia. Blood..

[16] Richardson S., Hirsch J.S., Narasimhan M., Crawford J.M., McGinn T., Davidson K.W., Northwell COVID-19 Research Consortium (2020). Presenting Characteristics, Comorbidities, and Outcomes Among 5700 Patients Hospitalized with COVID-19 in the New York City Area. JAMA.

[17] Caillot D., Couaillier J.F., Bernard A., Casasnovas O., Denning D.W., Mannone L., Lopez J., Couillault G., Piard F., Vagner O. (2001). Increasing volume and changing charac-teristics of invasive pulmonary aspergillosis on sequential thoracic computed tomography scans in patients with neutropenia. J Clin. Oncol..

[18] D’Haese J., Theunissen K., Vermeulen E., Schoemans H., De Vlieger G., Lammertijn L., Meersseman P., Lagrou K., Maertens J., Meersseman W. (2012). Detection of Galactomannan in Bronchoalveolar Lavage Fluid Samples of Patients at Risk for Invasive Pulmonary Aspergillosis: Analytical and Clinical Validity. J. Clin. Microbiol..

[19] Meijer E.F.J., Dofferhoff A.S.M., Hoiting O., Buil J.B., Meis J.F. (2020). Azole-Resistant COVID-19-Associated Pulmonary Aspergillosis in an Immunocompetent Host: A Case Report. J. Fungi.

[20] Koehler P., Bassetti M., Chakrabarti A., Chen S.C.A., Colombo A.L., Hoenigl M., Klimko N., Lass-Flörl C., Oladele R.O., Vinh D.C. (2021). Defining and managing COVID-19-associated pulmonary aspergillosis: The 2020 ECMM/ISHAM consensus criteria for research and clinical guidance. Lancet Infect. Dis..

[21] Ullmann A.J., Aguado J.M., Arikan-Akdagli S., Denning D.W., Groll A.H., Lagrou K., Lass-Flörl C., Lewis R.E., Munoz P.E., Verweij P. (2018). Diagnosis and management of Asper-gillus diseases: Executive summary of the 2017 ESCMID-ECMM-ERS guideline. Clin. Microbiol. Infect..

[22] Jenks J.D., Mehta S.R., Hoenigl M. (2019). Broad spectrum triazoles for invasive mould infections in adults: Which drug and when?. Med. Mycol..

